# Children with developmental language disorder: a frequency following response in the noise study

**DOI:** 10.1016/j.bjorl.2021.01.008

**Published:** 2021-03-02

**Authors:** Trandil H. Elmahallawi, Takwa A. Gabr, Mohamed E. Darwish, Fatma M. Seleem

**Affiliations:** a Tanta University Hospitals, Otolaryngology Head and Neck Surgery Department, Audiovestibular Unit, Tanta, Egypt; b Kafrelsheikh University Hospitals, Otolaryngology Head and Neck Surgery Department, Audiovestibular Unit, Kafrelsheikh, Egypt; c Tanta University Hospitals, Otolaryngology Head and Neck Surgery Department, Phoniatrics Unit, Tanta, Egypt

**Keywords:** Language development disorders, Evoked potentials, Brain stem, Speech perception, Noise

## Abstract

**Introduction:**

Children with developmental language disorder have been reported to have poor temporal auditory processing. This study aimed to examine the frequency following response.

**Objective:**

This work aimed to investigate speech processing in quiet and in noise.

**Methods:**

Two groups of children were included in this work: the control group (15 children with normal language development) and the study group (25 children diagnosed with developmental language disorder). All children were submitted to intelligence scale, language assessment, full audiological evaluation, and frequency following response in quiet and noise (+5QNR and +10QNR).

**Results:**

Results showed no statically significant difference between both groups as regards IQ or PTA. In the study group, the advanced analysis of frequency following response showed reduced F0 and F2 amplitudes. Results also showed that noise has an impact on both the transient and sustained components of the frequency following response in the same group.

**Conclusion:**

Children with developmental language disorder have difficulty in speech processing especially in the presence of background noise. Frequency following response is an efficient procedure that can be used to address speech processing problems in children with developmental language disorder.

## Introduction

Developmental language disorder (DLD) is the new term to replace specific language impairment (SLI). Children with DLD is a developmental disorder where a child with normal intelligence and hearing fails to develop language in an age-appropriate fashion. Its clinical diagnosis is based on the presence of a normal nonverbal IQ, evidence of expressive and/or receptive language significantly below expected for age and intelligence in addition to the absence of other specific conditions such as autism, global intellectual disability, metabolic or genetic disorders, or severe environmental deprivation.^1^ This condition was commonly called “specific” language impairment, however there is controversy as to how specific the condition is and whether the terminology should be changed to a more generic term, such as delayed language disorders (DLD). Ellis-Weismer et al.,^2^ Poor speech perception seems to be one of the primary deficits in children with DLD that might explain poor phonological development, impaired word production, and poor word comprehension.^3^ Genetic factors had been implicated in the occurrence of DLD, however, environmental factors play an important role.^4^ In addition, language processing requires good working memory capacity and linguistic abilities.^5^

Speech sounds are complex structures that are rich in harmonic structures, dynamic amplitude modulations, and multiple frequencies changing across the time course of the signal.^6^ Anatomically, the auditory brainstem is uniquely organized to encode rapid timing changes in auditory signals with exquisite accuracy that differentiates between tenths of milliseconds.^7^

Brainstem responses require adequate synchronization along with its neural structure which can be recorded in response to abrupt onset stimuli (e.g., click) or to speech stimuli which are more relevant to sounds we encounter in real life.^8^ Auditory brainstem response audiometry to speech stimuli is called frequency following response (FFR) and is found to be related to tonal language processing skills.^9^ FFR waves are composed of the transient of onset response represented by V-A complex, steady-state brainstem response (C, D, E, and F) and the offset response (O).^6^, ^10^ The transient response has been shown to encode the acoustic structure at the start of the voiced consonant-vowel stop syllables /ga/, /da/, /ba/.^8^ The steady-state brainstem response of FFR, on the other hand, is analyzed in the frequency domain and can be categorized into the envelope-following response (EFR) and the fine structure frequency-following response (FFR). The EFR spectral components occur as a result of the nonlinearities that are introduced by the rectification process of the speech envelope within the cochlea.^11^ It is commonly used to extract the evoked response that is phase-locked to the envelope of the speech stimulus which is modulated at the fundamental frequency F0. On the other hand, the FFR spectral content is generated as a result of auditory neural phase-locking that follows the fine structure of the speech stimulus and is used to extract the evoked response in the region of the first formant F1, and possibly the second formant F2 if it is sufficiently low in frequency to allow neural phase-locking.^12^

Children with DLD are known to have difficulty in perceiving speech and the condition may be worsened in the presence of noise. Noise degrades the acoustic input and requires more robust linguistic knowledge which is already impaired in those children as they have impaired cognitive abilities and attention deficits. In the presence of noise, children with DLD have to match between auditory input and linguistic memory representations from partial information.^13^ This theory will be addressed in this work using FFR. using advanced analysis of Matrix Laboratory software (MATLAB) in quiet and in noise.

## Methods

This work included 2 groups of children: control group (Group I); consisted of 15 children with normal language development) and study group (Group II) consisted of 25 children diagnosed with developmental language disorder.

The age range of children of both groups was 3–6 years. All children have a bilateral normal peripheral hearing (along with the frequency range of 250–8000 Hz) and bilateral normal middle ear function, their IQ was >85.

Children with DLD were diagnosed by speech-language therapist. Their diagnostic criteria include below-average language skills as expected for the child’s age, language abilities interfere with the child’s ability to communicate effectively with other people, the expressive language is characterized by non-specific words and short simple sentences to express meanings beyond the age at which children may be using more complex language in addition to difficulty in understanding of receptive language. Children with other difficulties such as attention deficit hyperactivity disorder (ADHD), dyslexia or speech sound difficulties were excluded from the study. Children with below-average IQ, or uncooperative children were also excluded from the study. This study was done at the audio-vestibular medicine unit, Kafrelsheikh University.

The methodology includes the following:

Basic audiological evaluation [pure tone audiometry, speech audiometry (GSI 61 audiometer, USA), immittancemetry (Interacoustic AT235 h, Denmark).

Intelligence Scale: using the Arabic version of the 5th edition of Stanford-Binet^14^ standardized by Faraj et al.^15^

Language assessment using modified Preschool language scale-4 (PLS-4) test.^16^

Auditory brainstem response audiometry (ABR) in response to click stimuli to verify the normal function of the neural pathway.

Frequency following response (FFR): in response to 40 ms/da/speech syllable presented at 80dBSPL, at repetition rate of 11.1 s. Analysis time was 75 msec including 15 ms pre-stimulus recording. FFR was recorded in quiet then in the presence of ipsilateral white noise at sound pressure level (SPL) of +5, +10 dB SPL (+5QNR and +10QNR).

The total number of sweeps was 1024, alternating polarity, the bandpass filter setting was 100–3000 Hz. Stimuli were presented monaurally via an Etymotic Research-3A (ER-3A) – insert shielded phone. Four disposable electrodes were placed at high frontal Fz (positive electrode), one low frontal Fpz (ground electrode). The last two electrodes were placed on the left and right mastoids (as a negative electrode or reference electrode) depending on the recording side. The electrode impedance was kept below 5-kOhm. Artifact rejection of ±31-μV was applied to reject epochs that contained myogenic artifacts and the gain factor was 100,000. Recording for FFR was done using Smart-Evoked potentials (Smart-EPs) of intelligent hearing system (IHS), USA. During data collection, the child was resting comfortably on the examination bed, watching a silent cartoon movie.

This work is compliant with the ethical standards and ethically approved from the institute for studies involving human beings or animals. The approval code is 30166/3/31.

Data analysis of FFR include the following:

Cross-correlation: Stimulus-response correlation was used to assess the degree of similarity between both using the FFR analysis module developed by Northwestern University. The onset of the response can be objectively determined in this manner. Before correlation, the stimulus was low-pass filtered (100–1000 Hz) to remove the consonant noise burst and allow higher correlations given the low-pass filter nature of the brainstem response.^17^ The response in the range of 20–40 ms was correlated with the sustained portion of the stimulus in the range of 13–34 ms as a function of the time shift between them at a given time displacement. The stimulus waveform was shifted in time to the response waveform (stationary) until the maximum correlation coefficient (r) was obtained. If the two signals were identical, r = 1. If the signals were identical but 180 degrees out of phase, r = 1 and if the signals were completely dissimilar, r = 0.^18^, ^19^ Maximum correlations were then converted to Fischer *z* scores for statistical purposes. Data collected from the control group acted as a baseline for comparison with the study group. Deviation of values outside the 95% confidence limits of control group data was considered abnormal.

Quiet-noise correlation: Responses in quiet and noise (+5 and +10 QNR) were cross-correlated to objectively quantify how much an individual is affected by the noise. When the correlation between the quiet and noise condition is high approaching 1, the noise is interpreted as having a minimal impact on the response, whereas lower correlations indicate that noise has a greater impact on the response^20^ Fisher’s transformation was used to convert the correlation values to z scores for statistical computations.

Fourier analysis: It was done to measure the raw amplitude values of F0, F1, and F2 frequency components of the response. Frequency encoding was analyzed using a Fourier analysis of two different time windows that include peaks D, E, and F of the response.

Cross phaseogram: It is used to capture the brain’s ability to discriminate between spectro-temporally dynamic speech sounds, such as stop consonants. The goal was to develop an analysis technique for ABR that taps into the sub-millisecond temporal precision of the response without relying on the subjective identification of individual response peaks.^21^ It was done to correlate between quiet and noise conditions for both sustained and transient responses. Thus, analysis is conducted at 2-time windows: transient response at 11–20 ms (for consonant–vowel transition) and sustained response at 21–40 ms (for steady-state vowels) aiming to determine whether or not there was noise-induced shift in phase or not. Phase shifts over the response spectrum frequency (70–1000 Hz) were calculated from transient and sustained response.^21^

### Statistical analysis

Statistical analysis included both Student's *t*-test and Mann Whitney's test for comparison of quantitative variables between two groups of normally and abnormally distributed data, respectively. The ANOVA test was used for comparison of quantitative variables between more than two groups of normally distributed data with Tuckey test as a post hoc test. Kruskal Wallis test was used for comparison of quantitative variables between more than two groups of not normal distributed data with Tamhane's test as a post hoc test. Chi-Square test (χ^2^) was used to study association between qualitative variables. Whenever any of the expected cells were less than five, Fischer's Exact test with Yates correction was used. Pearson correlation was used to show the correlation between two continuous normally distributed variables while Spearman correlation was used for not normally distributed ones. In all tests, *p*-value of  < 0.05 is considered statistically significant.

## Results

This study included two groups of children: the control group (GI): consisted of 15 children (4 males and 11 females). Their mean age was 5.04 ± 0.79 years. All children of this group had typical language development. As regard handedness, 13 (out of 15, 86.7%) were right-handed. The study group (GII) consisted of 25 children (19 males and 6 females). Their mean age was 4.77 ± 0.85 years and diagnosed with specific language impairment. There was no significant difference between both groups as regard age (*p* > 0.05).

Frequency following response (FFR) was done in 3 testing conditions: quiet, QNR of +5, and +10. Advanced analysis of FFR included the following:

### Stimulus-Response (S-R) correlations

In quiet (quiet correlation): Both groups showed similar positive correlation between stimulus and response in both ears which was significant (Table 1).

**Table 1:** Comparison between the right and left ears of control and study groups and comparison between both groups for Stimulus to Response (S-R) correlations.

	Ear	GI		GII		Mann Whitney test	*p*-Value
**Correlation coefficient (r)**	Right	0.097 ± 0.042		0.092 ± 0.028		0.18	0.85
	Left	0.096 ± 0.026		0.073 ± 0.052		1.45	0.14
		t = 0.08	*p* = 0.93	t = 0.58	*p* = 0.56		
**+10QNR**	Right	0.30 ± 0.16		0.41 ± 0.15		2.25	0.02
	Left	0.27 ± 0.15		0.35 ± 0.15		2.22	0.02
		u = 0.83	*p* = 0.40	u = 1.88	*p* = 0.06		
**+5QNR**	Right	0.40 ± 0.13		0.65 ± 0.10		4.30	<0.001
	Left	0.36 ± 0.14		0.60 ± 0.11		4.30	<0.001
		u = 0.99	*p* = 0.31	t = 1.39	*p* = 0.17		

*p significance < 0.05.

GI: control group; GII: study group.

In noise (Q-N correlation): Responses in noise at +5 and +10 QNR were cross-correlated for objective quantification of the effect of the noise on an individual. There was a low correlation between stimulus and response in both right and left ears in both groups suggesting a similar detrimental effect of noise. In each group, the noise effect was the same in the right and left ears, however, it has a more drastic effect on the study group than control in both ears at both ratios (Table 1).

**Figure 1:**
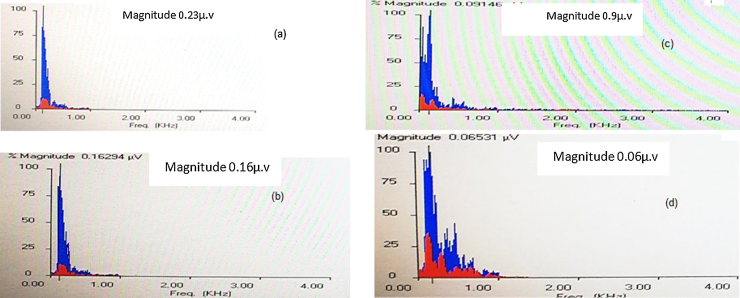
Fast Fourier Transform (FFT) that revealed the magnitude of amplitude of F0 and F1 in quiet in normal language developed child (a) and in a child with DLD (b) Typically developed language (TDL) at +5QNR in normal language developed child (c) and in a child with DLD (d).

Fast Fourier analysis: In this analysis, the amplitudes of the formant frequencies F0 and F1 were calculated and compared between the right and left ears at quiet and +5QNR conditions. Generally, F0 amplitudes were more robust than F1 amplitudes in quiet and noise condition in both groups. In quiet condition, the control group showed no differences between both ears, however, the study group showed significantly higher F0 and F1 amplitudes in the right ear when compared with the left. At +5QNR, the study group showed significantly higher F0 and F1 amplitudes in the right ear when compared with the left. The comparison between both groups showed significant higher F0 and F1 amplitudes in the control group in quiet condition only (t = 2.2 and *p* = 0.02). In each group, a comparison had been made between quiet and noise conditions and showed a significant reduction of F0 and F1 amplitudes in +5QNR recording condition when compared with quiet in both groups (Fig. 1, Table 2, Table 3).

**Table 2:** Comparison of the amplitudes of formant frequencies of F0 and F1 between the quiet and +5QNR conditions in both ears of both groups.

	GI				GII		
	Right ear		Left ear		Right ear	Left ear	
**F0 Hz**	Quiet	0.023 ± 0.005	0.029 ± 0.03	u = 1.48	0.019 ± 0.004	0.016 ± 0.004	t = 2.33 *p* = 0.02
				*p* = 0.13			
	+5QNR	0.010 ± 0.002	0.008 ± 0.002	t = 3.08	0.011 ± 0.002	0.008 ± 0.002	t = 4.40
				*p* = 0.051			*p* < 0.001
		t = 8.61	u = 4.68		t = 8.72	t = 8.57	
		*p* < 0.001	*p* < 0.001		*p* < 0.001	*p* < 0.001	
**F1 Hz**	Quiet	0.014 ± 0.002	0.013 ± 0.003	t = 1.05	0.012 ± 0.002	0.009 ± 0.001	t = 4.52
				*p* = 0.29			*p* < 0.001
	**+**5QNR	0.006 ± 0.001	0.005 ± 0.001	t = 1.81	0.006 ± 0.001	0.004 ± 0.001	t = 3.91
				*p* = 0.08			*p* < 0.001
		t = 9.94	t = 7.39		t = 10.99	t = 11.39	
		*p* < 0.001	*p* < 0.001		*p* < 0.001	*p* < 0.001	

*p significance < 0.05.

GI: control group; GII: study group.

**Table 3:** Comparison of the transient and sustained response amplitudes at both QNRs between right and left ears in both groups.

		GI		GII	
		Right	Left	Right	Left
**+10QNR**	Transient	0.19 ± 0.08	0.27 ± 0.15	0.30 ± 0.17	0.20 ± 0.11
		t = 1.31		u = 2	
		*p* = 0.18		*p* = 0.04	
	Sustained	0.19 ± 0.09	0.23 ± 0.10	0.22 ± 0.07	0.16 ± 0.05
		t = 0.97		u = 3.45	
		*p* = 0.32		*p* = 0.001	
**+5QNR**	Transient	0.26 ± 0.13	0.23 ± 0.10	0.64 ± 0.09	0.56 ± 0.08
		t = 0.87		u = 3.2	
		*p* = 0.38		*p* = 0.002	
	Sustained	0.25 ± 0.11	0.28 ± 0.24	0.55 ± 0.10	0.50 ± 0.15
		t = 0.33		u = 1.3	
		*p* = 0.73		*p* = 0.19	

*p significance < 0.05.

GI: control group; GII: study group; +10QNR: +10 Quiet to noise ratio; +5QNR: +5 Quiet to noise ratio.

Cross phasogram correlations: The comparison of the average phase shift of the transition and sustained regions of FFR in presence of noise relative to quiet in both right and left ears were done. The control group showed no significant effect of noise in both ears. Meanwhile, the study group showed a statistically significant higher phase shift in the right ear in the 2 testing conditions (+5QNR and +10QNR) in both transient and sustained responses, however, it did not reach a significant level at +5QNR in the sustained response. The comparison between control and study groups showed: at +10QNR, the transient response showed no significant difference between both groups in either right or left ears, while the sustained response showed a higher shift in the left ear. At +5QNR, the transient and sustained responses showed a significant higher phase shift in the study group in both ears (Table 4).

**Table 4:** Comparison of the transient and sustained response amplitudes at both QNRs between right and left ears between both groups.

	Ear	GI	GII	Mann Whitney test	*p*-Value
**+10QNR**					
Transient	Right	0.19 ± 0.08	0.30 ± 0.17	1.79	0.07
	Left	0.27 ± 0.15	0.20 ± 0.11	1.20	0.23
Sustained	Right	0.19 ± 0.09	0.22 ± 0.07	1.40	0.16
	Left	0.16 ± 0.10	0.23 ± 0.05	2.00	0.04
**+5QNR**					
Transient	Right	0.26 ± 0.13	0.64 ± 0.09	5.24	<0.001
	Left	0.23 ± 0.10	0.56 ± 0.08	5.24	<0.001
Sustained	Right	0.25 ± 0.11	0.55 ± 0.10	5.11	<0.001
	Left	0.28 ± 0.24	0.50 ± 0.15	3.90	<0.001

*p significance < 0.05.

## Discussion

The brainstem response to speech syllables (FFR) can be used as a neural index of asynchrony in children with DLD. The primary aim of this study was studying FFR in children with DLD and how FFR could contribute to the understanding of speech sounds processing at brainstem level in normal-hearing children and those with DLD.

In this study, FFR was recorded with normal morphology in children with DLD, however, with delayed peak timing supporting the previous notion of Banai et al.^22^ Several parameters of FFR were studied starting with the stimulus-response (S-R) correlations both in quiet and in noise conditions. In quiet, where there was a good positive correlation between stimulus and response identified. However, in noise, there was a poor correlation between stimulus and response in either the right or left ears of both groups. These findings suggest a similar detrimental effect of noise which was more pronounced in children with DLD. Robertson et al.^23^ and Basu et al.^24^ explained poor speech perception in noise in those children with DLD to result from failure of the phonological abilities that underlie language, or failure of cognitively-based processes such as attention, and memory that do play a substantial role in speech perception in noise.^3^, ^25^ Additional important factors such as age and maturation had been reported by Wilson et al.^26^ and Myhrum et al.^27^ The authors emphasized the gradual maturation of binaural processing and the maturation of other cognitive abilities that may be involved in speech perception in noise, such as attention and processing speed, in addition to the language development that happens during this period.

Another FFR parameter was calculating the amplitudes of the formant frequencies F0 and F1 using Fast Fourier analysis. There was a significant higher F0 and F1 amplitudes in the right ear when compared with the left in both quiet and noise conditions in the study group. These findings suggested the presence of right ear advantage in children with DLD that is apparent at the brainstem level. This could be explained by faster central conduction time as a result of more direct access of the right ear to the left hemisphere through the contralateral pathway which is the main auditory pathway.^28^ Both groups of children showed significant higher F0 and F1 amplitudes in quiet than in noise conditions, emphasizing the drastic effect of noise on speech encoding in both groups of children. During their processing, speech sounds are filtered into bands of narrowband waves which are further decomposed into fast fluctuating temporal fine structures (TFSs) and slowly varying envelopes (ENVs).^29^ The impairment noticed in children with DLD might suggest a difficulty in the detection of both components^30^, ^31^ that might start at the level of the auditory brainstem and indicated that the representation of the fundamental frequency (F0) differs between children with DLD and normal children.^24^ Thus, losing such temporal precision in the subcortical encoding of sounds leads to difficulty in speech perception particularly in the presence of noise.

Pitch encoding is another contributing factor to speech perception. It is dependent on F0 and its harmonics and reflects neurobiological processes at both cortical^32^ and subcortical levels.^33^ The F0 and the lower harmonics provide essential acoustic cues and pitch cues, along with spatial, timing, and harmonic cues aid in sound identification particularly in the presence of noise.^34^ Andreson et al.,^35^ reported that the strength of subcortical encoding of pitch (determined by the low harmonics) is an important factor for successful hearing in noise. The neural mechanism responsible for such observation operated exclusively in response to the time-varying formant transition period, a region particularly important for syllable identification and is most vulnerable to masking by background noise.^24^ So, impaired pitch perception may be a factor in the speech perception deficits in noise experienced by children with DLD. Cognitive processes such as attention, memory, and object formation likely also affect the subcortical encoding of sound. When a listener judges a signal as important, auditory attention works to extract relevant information from the competing background noise and stores them in working memory.^36^ The cortex then uses this information to make predictions of the most relevant features of the stimulus with subsequent corticofugal enhancement in lower subcortical structures that provide improved signal quality to the auditory cortex. Thus, children with poor speech perception in noise likely have a deficient encoding of sound due to a failure of cognitively based processes (attention, memory) in addition to the noise effect on the subcortical sensory function.^31^, ^37^ Normal children can exclude signal from background noise due to their full language maturation that leads to effective speech stream and normal top-down cognitive function.

FFR provides brainstem representation of pitch-related spectral amplitudes in a repetitive stimulus context. The greater this representation, the better speech perception in noise. The ability to modulate representation of pitch based on stimulus regularities is important for “tagging” relevant speech features, a key component of object identification and speech perception in noise. Children with diminished F0 and lower harmonics representation have difficulties with pitch perception and subsequent auditory grouping and are likely to have a reduced ability to lock onto the pitch of the target signal, contributing to poorer speech perception in noise.^35^ This study also showed that F0 was more robust than F1 either in quiet or noise conditions in both groups supporting the earlier work on the differential susceptibility to the noise of the F0 and F1 components of the FFR. This is consistent with Laroche et al.^38^ who reported that in speech processing, the pitch of resolved harmonics and that of unresolved harmonics are processed in different but interacting pathways that converge in the upper brainstem.^39^

Cross phasogram correlations were done to compare the average phase shift of the transition and sustained regions of FFR in noise relative to quiet. There was no phase shift between right and left ears in normal children, however, children with DLD showed a significant phase shift at the two noise conditions (+5QNR and +10QNR) except the sustained response at +5QNR. The comparison between both groups showed that, at +10QNR, the transient response showed no significant difference between both groups in either right or left ears. However, the sustained response showed a higher shift in the left ear. At +5QNR, the transient and sustained responses showed a significant higher phase shift in both groups in both ears. The comparison between control and study groups showed higher sustained response shift in the left ear at +10QNR only. However, at +5QNR; both the transient and sustained responses had significantly higher phase shift in children with DLD in both ears. These findings indicated the higher the noise level, the higher the impact on speech processing in children with DLD. Speech discrimination in noise is correlated with transient response timing and harmonic encoding of speech presented in background noise^40^ and it was noticed that cortical response degradation in noise is greatest for children with delayed responses in the brainstem.^41^ This suggested that subcortical encoding deficits seen for children with DLD extend to affect both the fast temporal (transient) and dynamic spectrotemporal elements of the signal which are crucial for distinguishing speech sounds.^24^

## Conclusion

The current study provides more evidence that children with DLD have deficits in speech perception especially in the presence of background noise. FFR can be used efficiently for the assessment of speech sounds processing at the brainstem level and in those children it showed their reduced ability to lock onto the pitch of the target signal. Also, their impaired cognitive functions such as attention and memory worsened the situation, which might improve with maturation and language development. FFR can be added to the behavioral test battery used for assessment of speech perception in children with DLD, especially the young age groups. It might also help in designing the rehabilitation programs that should be tailored for each child.

## Conflicts of interest

The authors declare no conflicts of interest.
